# Personalized Nutrition and the Regulatory Framework – Moving Forward

**DOI:** 10.1016/j.advnut.2025.100383

**Published:** 2025-01-24

**Authors:** Andrew A Bremer

**Affiliations:** Office of Nutrition Research, National Institutes of Health, Bethesda, MD, United States

Precision and personalized nutrition (PN) aim to leverage human variability to design tailored dietary interventions to improve health [1]. Although there are no standardized definitions of precision nutrition and PN, and the terms are often used interchangeably, precision nutrition can be considered to focus on specific subgroups within the general population and to provide tailored dietary recommendations based on deep phenotyping technologies utilizing high-throughput -omcs approaches and integrating information at scale; alternatively, PN focuses on the individual at a personal level, tailoring dietary recommendation based on unique genetic, phenotypic, medical, and lifestyle information. Regardless, the goal of both approaches is the same—to provide targeted advice to an individual to preserve or ameliorate health and well-being using dietary interventions that leverage human variability [1]. Importantly, the evidence base used to inform both approaches is also the same—multidisciplinary in nature (relying on nutrition, systems biology, and behavioral sciences) and rapidly evolving with technological advances. New biomarkers continue to be discovered, innovations in wearables and other noninvasive devices continue to increase the amount of real-time data provided, and advances in artificial intelligence (AI) and machine learning (ML) models continue to refine their ability to generate personalized recommendations for lifestyle-behavior changes.

There is also increasing consumer demand for tailored health interventions [2], underscoring the importance of developing scientifically robust, inclusive, and scalable PN programs. Yet, as the PN field grows, it is critical to also consider the regulatory framework under which it operates to maintain its credibility, build user trust and engagement, and prevent misinformation. Advancements in the PN field are also raising important questions, particularly with respect to user safety, security, health, transparency, and privacy. To address these issues, a virtual workshop hosted by the Personalized Nutrition Initiative at the University of Illinois was held in March 2024. The workshop goals centered around the current regulatory framework for PN and focused on addressing the regulatory implications of current PN programs, future innovations within the current United States (US) regulatory framework, and existing complexities of oversight; the main purpose of the workshop was to better understand current regulatory guidelines and how PN innovations fit within them to help guide responsible program development. The outputs of the workshop are highlighted by Donovan et al. [3] in this issue of *Advances in Nutrition*.

Specifically, the areas of food, dietary supplements, in vitro diagnostics, and medical wellness devices were discussed, as were the topics of new emerging devices, biomarkers, behavior-based tools, and the integration of AI/ML into PN programs. Importantly, because PN programs typically combine many of these topic areas, the potential need to differentially regulate these multiple components was also considered. As Donovan et al. [3] report, the workshop participants concluded that regulatory guidance for PN programs should focus on the following: *1*) the safety and accuracy of tests and devices; *2*) the credentials of the experts developing PN advice; *3*) responsible and clear communication of information and benefits; *4*) substantiation of scientific claims; and *5*) procedures to protect user privacy. They also note that as the PN field evolves, the need to adapt the existing regulatory framework may need to be considered and that a close relationship between the academic and regulatory sectors is both an opportunity and requisite to provide users with transparency, build trust, and create a source of differentiation for PN innovators.

The topic raised by Donovan et al. [3] (i.e., the intersection of science with regulatory decision making) could not be more timely, as exemplified by a recent joint NIH-US Food and Drug Administration Nutrition Regulatory Science Workshop (held in December 2024) focused on how nutrition science can generate evidence and data to inform food-related policy and regulatory decision making [4]. Examples of topics discussed at the joint NIH-US Food and Drug Administration workshop include ultraprocessed foods, impact analysis and implementation science related to regulatory actions, and emerging technological innovations related to nutrition regulatory science. Although PN as a field was not discussed as a standalone topic per se, the Nutritionfor Precision Health, powered by the All of Us Research Program [5] was highlighted in a session of the workshop devoted to “Technical Innovations and Other Research Opportunities to Inform Nutrition Regulatory Science.” Launched in 2023, the goal of the Nutrition for Precision Health Program—the largest precision nutrition research effort of its kind—is to use AI to develop algorithms that predict individual responses to foods and dietary patterns; tiered levels of data are expected to be available to the public in 2027. Additional topics related to PN discussed in the session included the following: *1*) innovative advancements in the field of dietary assessment; *2*) -omics data and ML; and *3*) applying AI/ML to study dietary patterns and health outcomes.

Importantly, Donovan et al. [3] also highlight the value of collaborative multidisciplinary teams in the implementation of PN programs—including but not limited to dietitians, physicians, nurses, psychologists, behavioralists, and health and wellness coaches. This point cannot be overemphasized, especially given the need to increase the number of investigators and providers in many of these disciplines [[6], [7], [8], [9]]. Fortunately, to address these needs, training programs specifically focused on the integration of nutrition with AI, ML, systems biology, systems science, Big Data, and computational analytics were launched by the NIH in 2023 [10]. Ultimately, the goal of these NIH training programs is to enhance the development of a research workforce with advanced competencies in AI, ML, and data science analytics to develop innovative solutions to address issues related to nutrition and diet-related conditions.

In summary, Donovan et al. [3] nicely outline how PN can operate within the current US regulatory framework. Importantly, they also present challenges, opportunities, and potential solutions for the future of PN and suggest that the regulatory environment for PN programs may need to evolve with this rapidly advancing market to ensure the delivery of safe, effective, substantiated, clearly communicated, and accessible solutions. Given the consumer demand for PN, the time is now to move the field forward in a way the public can trust.

## Author contributions

The sole author is responsible for all aspects of this manuscript.

## Funding

The author reports no funding received for this work.

## Conflict of interest

AAB is an Editor for Advances in Nutrition. He did not directly peer-review the manuscript.
